# Editorial: Epigenetics of the immune component of inflammation

**DOI:** 10.3389/fimmu.2022.1000836

**Published:** 2022-08-22

**Authors:** Yan-Jun Liu, Haitao Wang, Hai-Jing Zhong, Cheong-Meng Chong, Guan-Jun Yang

**Affiliations:** ^1^ State Key Laboratory for Managing Biotic and Chemical Threats to the Quality and Safety of Agro-products, Ningbo University, Ningbo, China; ^2^ Laboratory of Biochemistry and Molecular Biology, School of Marine Sciences, Ningbo University, Ningbo, China; ^3^ Key Laboratory of Aquacultral Biotechnology Ministry of Education, Ningbo University, Ningbo, China; ^4^ Thoracic Surgery Branch, Center for Cancer Research, NCI, NIH, Bethesda, MA, United States; ^5^ College of Pharmacy, Jinan University, Guangzhou, China; ^6^ State Key Laboratory of Quality Research in Chinese Medicine, Institute of Chinese Medical Sciences, University of Macau, Macao, Macao SAR, China

**Keywords:** epigenetic regulation, immune component, inflammation, post-translational modifications, DNA modification, non-coding RNA regulation

Epigenetics is a type of inheritance that involves the change of the expression and function of genes but without changes in the DNA sequence (1, 2). Mounting evidence revealed that epigenetic modifications (DNA/RNA modifications, non-coding RNAs, and post-translational modifications) are linked with the genesis of inflammation *via* modulating a variety of immune components such as cytokines, transcription factors, complements, pattern recognition receptors, and other inflammation-related genes. The epigenetic genes are constitutively activated or deactivated in multiple immune- or non-immune cells under inflammatory conditions and contribute to a variety of inflammatory diseases, such as rheumatoid arthritis (RA) [Yang et al., as well as diabetes (3), Ding et al.], and cancers (4, 5), etc. Inflammation often hijacks various epigenetic mechanisms to promote occurrence and development of these diseases (6, 7). In turn, epigenetic modifications also mediate pathologic development of inflammation by regulating immune components in inflammatory microenvironments (8, 9). Numerous preclinical and clinical studies showed that epigenetic modulators, including KDM5 inhibitors (10), LSD1 inhibitors (11), HDAC inhibitors (12), EZH2 inhibitors (13, 14), and BET inhibitors (4, 5, 15) exhibit anti–inflammatory activities *in vitro* and *in vivo*. Therefore, exploring the roles of epigenetics of immune components in inflammation not only helps us to understand the progress of inflammatory diseases, but also contributes to developing novel strategies for diagnosis and treatment of these diseases.

This Research Topic contributes to a better understanding of epigenetics in the immune component of inflammation and highlights the clinical significance of epigenetic regulation in disease diagnosis and drug discovery. This Research Topic accepted a total of 42 articles from 285 authors, demonstrating great interests in this field. This topic can be divided into the following subtopics:

## DNA/RNA modifications

The hypermethylation in the promoter regions of inflammatory genes often leads to their inactivation and suppresses inflammatory diseases. Conversely, the hypomethylation in the cis–acting elements of these genes upregulates their levels and contributes to inflammation (16). In this Research Topic, Bordagaray et al. found that the promoter of *TLR2* gene in peripheric mononuclear blood cells (PBMCs) from patients with apical periodontitis (AP) has much higher global methylation than that of controls, suggesting that hypermethylation is responsible for sustained systemic inflammation in AP. Zhao et al. summarized the roles of DNA methylation of T cell related genes in the development and differentiation of T lymphocytes, and the importance in diagnosis and drug action. Guo et al. determined the molecular signatures associated with meningitis induced by *Glaesserella parasuis* based on methylome and transcriptome–based integration analysis, which provides potential diagnostic biomarkers and therapeutic targets for *G. parasuis* induced pig meningitis. DNA methylation also contributes to inflammation induced tumorigenesis (17). Yang et al. summarized the emerging roles of DNA methylation–mediated inflammation in tumorigenesis of non–small cell lung cancer (NSCL) and found that targeting these cell events is a potential strategy for NSCL treatment. Chen et al. also considered that the DNA methylation regulation of inflammation is the key factor for tumor microenvironment (TME) in glioblastoma. Based on DNA methylation–driven genes based prognostic model, Tian et al. identified five target genes and five agents with therapeutic potentials for high–risk breast cancer (BC) patients. Further *in vitro* analysis indicated that (+)–JQ1 is the best candidate agent for BC treatment among them. Yang et al. identified and characterized extrachromosomal circular DNAs (eccDNAs/ecDNAs) in placentas with fetal growth restriction (FGR) based on circle–seq and Gene Ontology (GO) and Kyoto Encyclopedia of Genes and Genomes (KEGG) pathway enrichment analysis, and shed light on the formation mechanisms and the networks with noncoding RNAs (ncRNAs), which provides a new vision for the screening of new biomarkers and therapeutic targets for FGR.

N6–methyladenosine (m6A) is a common RNA modification mediating inflammation–related diseases *via* regulating genetic expression at the post–transcriptional level (18). Guo et al. comprehensively evaluated the correlation between N6–methyladenosine (m6A) regulators and the immune microenvironment in pancreatic cancer (PC) *via* integrating analysis of seven PC databases, and found that these samples can be divided into two clusters by consensus clustering for m6A regulators. The results showed that patients with lower m6A regulators tend to have higher immune cell infiltration and a better survival rate. Meanwhile, they also constructed risk scores using the least absolute shrinkage and selection operator (LASSO) regression analysis based on the expression of 16 m6A regulators in the reorganized training cohort to help precisely predict PC patient prognosis and immunotherapy benefits. The results demonstrated that six m6A regulators could be used as a prognostic signature to assess sensitization to immune checkpoint inhibitor and overall survival (OS) of PC patients, and patients with low–risk scores were associated with higher response to immunotherapy and a longer OS. Du et al. also found similar functions of m6A regulators in acute myeloid leukemia. Apart from coding RNAs, m6A also could modify ncRNAs and regulate inflammation (19). Xu et al. found that m6A modified cirRNA promoted the injury of MAC–T cells infected by *Staphylococcus aureus* and *Escherichia coli via* CircRNA–miRNA–mRNA interaction networks, suggesting m6A–modified CircRNA is a potential target for mastitis and other inflammatory diseases. Wu et al. comprehensively analyzed the correlations among the expression of m6A–related LncRNA, TME, and OS of osteosarcoma, the results showed that m6A–related long ncRNAs (lncRNAs) is positively correlated with occurrence and mediates TME remodeling in osteosarcoma *via* infiltrating immune cells, indicating m6A–related lncRNAs could be used as a biomarker to predict patient prognosis and target cancer therapy.

## Noncoding RNAs

NcRNAs including microRNAs and lncRNAs are another kinds of epigenetic modes, which could also regulate various inflammatory events (20). Rodríguez–Muguruza et al. found that a panel of exomiR–25–3p, exomiR–0451a, and soluble TWEAK could be used as biomarker for early diagnosis of RA. Wang et al. verified that miR–382 promoted M2–like macrophage (Mф) polarization *via* activating SIRP–α/STAT3 signaling in aristolochic acid–induced renal fibrosis, suggesting that miR–382 is a critical regulator for M2–like Mф polarization and a promising therapeutic target for renal fibrosis. Gu et al. reviewed the biosynthesis and functions of miR–233 in innate immunity, summarized the roles of miR–233 in liver physiopathology and prospected the therapeutic strategies. Jiang et al. outlined the roles of miRNAs–mediated inflammation in wound healing and considered that miRNAs could be a potential therapeutic target for wound healing. Xu et al. explored the mechanisms regulating dysfunctions of immune cells and inflammatory phenotypes in ulcerative colitis (UC) based on several bioinformatics analysis, and identified XIST, miR–9–5p, miR–129–5p, and miR–340–5p as the potential therapeutic targets for UC. Gong et al. performed the small RNAs and miRNAs profiling and integrative analysis of chronic epididymitis (CE) and identified a regulatory network containing 22 miRNAs and 31genes, which would contribute to improving the understanding of the roles of miRNA–mRNA in the pathogenesis of CE and provide molecular candidates for the development of potential biomarkers for human CE. LncRNA, an emerging epigenetic modification, has been found to mediate varieties of inflammatory diseases (21). Jiang et al. showed that LncRNAs could modulate Mф polarization through pro–inflammatory or anti–inflammatory mechanisms and thus mediate the process of inflammation–assocaited diseases such as infection, cancer, autoimmune diseases, and metabolic diseases. Guo et al. outlined the TEM–regulated functions of LncRNAs *via* mediating Mф polarization, neutrophil recruitment, T cells functions, and NK cells cytotoxicity, and contributed to tumorigenesis, angiogenesis, and cancer metastasis. Moreover, they summarized the prospective importance of LncRNAs as cancer prognostic biomarkers and therapeutic targets in clinic.

## Post–translational modifications

Post–translational modifications such as methylation/demethylation, acetylation, O–GlcNAcylation, and phosphorylation are epigenetic mechanisms potentially orchestrating the activation or inactivation of inflammation (Lin et al.). Lin et al. summarized the various roles of histone modifications in inflammatory diseases, indicating potential diagnostic biomarkers and therapeutic targets for these diseases. Xu et al. introduced the roles of phosphorylation in modulating inflammatory cell death, which suggested some kinases or phosphatases–mediated inflammatory diseases are potential target for inflammation–related diseases. Ouyang et al. summarized the effects of O–GlcNAcylation on tumor–associated inflammation and the mechanisms of O–GlcNAc–mediated inflammation in tumorigenesis, which provides a theoretical basis for the development of anti–cancer agents acting against inflammatory tumors by targeting O–GlcNAcylation. A chronic inflammation triggered methylation/demethylation switch is involved in tumor initiation and progression (22, 23). Li et al. found that EZH2 inhibitors GSK126 and EPZ6438 exhibited their anticancer activities in colorectal cancer (CRC) *via* directly suppressing proliferation of CRC cells, and promoting Mф polarization to tumor–suppressive M1 Mф. Yang et al. summarized the roles of JMJD histone demethylases in the crosstalk between inflammation and cancer and highlighted the potential applications of modulating cancer–related inflammation *via* targeting JMJD histone demethylases for cancer therapy. Wang et al. focused on the structure, biological function, and potential application of JMJD6 in tumor immune regulation and targeted therapy. Acetylation is also an important epigenetic modification involved in multiple inflammatory diseases (Lin et al.). Zhu et al. demonstrated that histone deacetylase 3 (HDAC3) activated NF–κB signaling *via* p65 deacetylation and promoted local prostaglandin E2 (PGE2) production in activated–microglia from a damaged cortex. HDAC3–mediated PGE2 production by microglia promoted phobic anxiety susceptibility after stroke, suggesting targeting HDAC3 might be a candidate for new therapy. Bromodomain and extra–terminal domain (BET) proteins are important in several inflammatory diseases (4, 5). Our studies found that acetylation reader BRD4 inhibitor 13a could eliminate NF–κB–driven triple–negative breast cancer (TNBC) cells *via* blocking the interaction BRD4 and acetylated RelA at lysine–310 site (5). Lei et al. revealed that BRD4 also modulated Histone 3 acetylation *via* FGFR2–BRD4 axis, and BRD4 inhibitors exhibited synergistic effect with immune check blockade in TNBC therapy. O’Connor et al. demonstrated that BET inhibitor (+)–JQ1–treatment reduced LPS–induced chromatin remodeling and suppressed the expression of inflammatory genes in Mф, and alleviated microbiota–dependent colitis compared with vehicle–treated mice.

## Author contributions

All authors listed have made a substantial, direct, and intellectual contribution to the work and approved it for publication.

## Funding

This work is supported by the National Natural Science Foundation of China (31972821), the General Scientific Research Project of Education of Zhejiang Province (Y202147351), the Starting Research Fund of Ningbo University (422210113), the Natural Science Foundation of Guangdong Province (2021A1515012520), the Science and Technology Development Fund, Macau S.A.R (FDCT)(0071/2021/A). Guangzhou Basic and Applied Basic Research Foundation (33121073) and the Fundamental Research Funds for the Central Universities (11620355).

## Acknowledgments

I would like to extend my sincere thanks to the guest editorial team and all the reviewers who participated in the handling of this topic. At the same time, I would like to express my sincere thanks to the authors who contributed excellent works to this topic.

## Conflict of interest

The authors declare that the research was conducted in the absence of any commercial or financial relationships that could be construed as a potential conflict of interest.

## Publisher’s note

All claims expressed in this article are solely those of the authors and do not necessarily represent those of their affiliated organizations, or those of the publisher, the editors and the reviewers. Any product that may be evaluated in this article, or claim that may be made by its manufacturer, is not guaranteed or endorsed by the publisher.
